# Disparity in Perceptions of Social Values for Ecosystem Services of Urban Green Space: A Case Study in the East Lake Scenic Area, Wuhan

**DOI:** 10.3389/fpubh.2020.00370

**Published:** 2020-09-29

**Authors:** Yuanyuan Chen, Xinli Ke, Min Min, Peng Cheng

**Affiliations:** College of Public Administration, Huazhong Agricultural University, Wuhan, China

**Keywords:** social value, ecosystem service, urban green space, SolVES, environmental conditions

## Abstract

Urban green space can bring various ecosystem benefits to diverse social groups. Among those ecosystem benefits, intangible social values are often neglected but highly relevant to human welfare. Existing research on the social values of urban green space often focusses on the perspective of urban inhabitants rather than tourists, even though tourists are also major beneficiaries. By combining different data sources into a comprehensive source about green-space social values, we investigated the disparity between inhabitants' and tourists' perceptions about space-associated social values, and further explored the underlying environmental conditions in the East Lake scenic area, Wuhan. For this, we collected 347 questionnaires through an on-site survey and 11,908 photos uploaded by 2165 social media users (Sina Blog), and we used SolVES (Social Value for Ecosystem Services) to uncover the spatial patterns of social values and the relationships between social value indicators and natural surroundings. Social-value hotspots occurred near water and trails. Perceptions differed, however, between inhabitants and tourists. Inhabitants perceived a larger scale of social values and could benefit more from recreation and economic values. Tourists, on the other hand, showed greater appreciation for aesthetic and cultural values. Environmental features were associated with social values to differing extent; distance to water and land use/cover exerted significantly influence. These findings should be taken into consideration to improve urban spatial planning and to optimize green infrastructures for human welfare.

## Introduction

Urban green space (UGS), as public and private open spaces in urban areas (1), can provide multiple kinds of service: water regulation, pollution reduction (2), noise alleviation (3), carbon storage (4), provision of habitat, and opportunities for cultural services (5). However, intangible and non-material benefits of UGS have received less attention in past research (6–8). Increasing evidence shows that the social values of ecosystem services could strengthen links between humanity and nature (9–12).

Research on subjective perceptions of UGS will provide a better understanding of the quality and quantity of urban green spaces (13, 14), as well as ecosystem services. Cultural ecosystem services provide the critical role of UGS from the perspective of users, and thus offer a promising way to integrate citizens' perceptions into urban planning (15, 16). Investigation of a pooled sample may inform policy makers by integrating perspectives and thus avoid interpretative difficulties due to stakeholder heterogeneity.

While it would be possible to examine the whole group at once, stratification of stakeholders delivers more detailed information about diverse interests (17); surfacing the diversity of perceptions can increase public managers' awareness of how different benefits are felt by different groups of people (18, 19). The groups are often distinguished by features such as familiarity with green infrastructure (20), gender and age (21), religion (7). Moreover, the spatial dimension of ecosystem service benefits is particularly important when considering important stakeholder groups (22). Existing research indicates that urban residents and visiting tourists conflict in their preferences over congestion, environmental protection, and employment opportunities (23, 24), and their knowledge of the area (25). An especially clear stratification of residents and tourists is hence found in coastal regions, forests (26) and national parks (27). As for UGS, previous research has focused mainly on benefits for the local residents who directly and perpetually benefit, and on investigating the market value for tourist and resident visitation (28–30). However, tourists' travel costs and familiarity differ markedly from urban residents' (31). Therefore, a systematic analysis has been employed to incorporate the residents' perspectives with tourists' perspective to investigate the social values of UGS by investigating how residents and tourists perceived non-material value in combination with inherent environment features.

Understanding the stakeholder perception involved a public participatory geographic information system (PPGIS) (9, 32, 33). PPGIS use is becoming increasingly widespread across ecosystem service assessment because the map makes information explicit, visualizable, and informative to urban planners during the decision process and during management of urban green spaces (34). Question-based and photo-based data are the main data sources for mapping and evaluating ecosystem services. Question-based data collection was obtained from face-to-face, telephone interview and on-line questionnaire interviewing (35, 36). Questionnaire responses directly reflect the perspective of different stakeholders at a specific time point. However, the questionnaire is inadequate for telling the whole story over a time period. Geo-tagged photos from employed visitors and social media compose photo-based data sources. Visitor-employed photography (VEP) can provide visitor trajectories, but are constrained by time consumption, expense and the spatial scale (37, 38). Social media platforms such as Flickr, Sina Blog, and Instagram can provide a large quantity of time series data at a lower cost than traditional surveys (39–41). Relevant studies focus on spatial and temporal trade-offs of ecosystem services (42), the impact of landscape change on ecosystem services (43), and human benefits under various scenarios (12). In particular, Sina Blog had as many as 550 million monthly active users in March 2020 (http://ir.weibo.com/), and is one of the most popular social media platforms in China. Until now, little work has been done coupling the questionnaires with social media. Integrative data sources across documenting users' experiences of green space over periods of time might offer interesting, new prospects for urban green space design and management.

Mapping of UGS services can be informative for urban planning and sustainable development (44). Less is known, however, about how to characterize and represent non-material values of ecosystem services in decision-making. Social values offer one way to evaluate the subjective, intangible services, especially for cultural services (45, 46). Social values originated from the evaluation of forest values (47). The individual value indicators composing the social-value typology (Table 1) provide the foundation for understanding the preferences of different stakeholder groups. Social Values of Ecosystem Services (SolVES), a GIS application from the United States Geological Survey (USGS), is based on that typology and has been proved useful for evaluating ecosystem services, trade-offs between value indicators, and social value of regions where data are unavailable (48–51). SolVES quantifies the relationships between social values, people's perceptions, and environmental conditions. By considering both the ecological and social contexts of values referring to ecosystem services, valuation results can be made meaningful for the spatial allocation of relevant ecosystem services and day-to-day decision making.

**Table 1:** Typology applied in on-site survey and classification of Sina Blog photos.

**Social value indicators**	**Value descriptions**
Aesthetic	I enjoy the beautiful scenery, sights, delightful sounds, etc.
Biodiversity	It provides a variety of fish, wildlife, plant life, etc.
Cultural	It is a place for me to continue and pass down the wisdom and traditions, participate various cultural activities.
Economic	It provides timber, fisheries, minerals, and/or tourism opportunities such as outfitting and guiding.
Future	It allows future generations to know and experience tradition, lifestyle right as them are now.
Historic	It has architectures and stories of natural and human history that matter to me, others, or the nation.
Intrinsic	I value it in and of itself, whether people are present or not.
Learning	We can learn about the environment through scientific observation or experimentation.
Life Sustaining	It is the habitat of creatures and helps preserve, clean, and renew air, soil, and water.
Recreation	It provides a place for my favorite outdoor recreation activities.
Spiritual	It is a special place to me or because I feel reverence and relaxation here.
Therapeutic	It provides a wonderful place for exercising and makes me feel stress free, physically and/or mentally.

*Adapted from Sherrouse et al. (49)*.

This paper investigates the spatial pattern of social values and examines the relationship between perceived social values and environmental conditions at the East Lake Scenic Area. Residents and tourists are the main stakeholders as mentioned above. Questionnaire and social media data were analyzed using SolVES to address three objectives: (1) discern the spatial pattern of perceived social values; (2) compare the social value indicators between two stakeholder groups; (3) identify relationships between preferable social values and environmental conditions. The paper aims to create space for discourse on the social values of urban green area and enlighten thought about how the perceptions of different visitor groups can be more effectively integrated into urban planning and green space management decisions.

## Methods

### Study Area

Wuhan (113.68–115.08E, 29.96–31.36N) is the transport and economic center of central China and the capital of Hubei Province, with a total area of 856,915 ha and a population of 108.93 million (52). The climate is subtropical monsoon, with an average temperature of 16°C and average precipitation of 1,200 mm. Rivers and lakes are the characteristic ecosystems of the area. The Chang Jiang (which becomes the Yangzi River downstream) and the Hanshui (the Yangzi's longest tributary) cross the city. The second largest inner-city lake in China is located in the East Lake (Dong Hu) scenic area (ELSA, 1320 ha), which is the well-known “National 5A-class tourist attraction.” Owing to its unique and essential location, the East Lake provides habitat suitable for over 500 species of flora and fauna. From 2000 to 2020, the government has proposed and implemented policies to improve the green elements of Wuhan (53). Notably, the urban greenway construction around the East Lake, with a total length of 101.98 km, connects eight parts of the ELSA: Mo mountain, Tingtao, Luoyan, Chuidi, Baima, Hou lake, Yuguang, and Yujia mountain. It significantly improves the environment and draws attention to its characteristics. Overall, 23 million visitors arrived in the ELSA in 2019, with an increase of 15.12% than the last year (54). Visitor expense in the ELSA contributes to local economies and supports human well-being by providing recreational opportunities, and promotes environmental stewardship. The ELSA demonstrates a good balance of ecological protection and financial benefit.

### Data Source

The collection of data comprised two phases: an on-site survey, and collection from social media. The on-site survey included both a pilot survey and a formal survey. The pilot survey (*n* = 20, response rate 100%) was conducted in December, 2018 to validate the questionnaire. The formal survey was collected from a representative sample of people over the age of 14 who visited ELSA during a high-use vacation period (Labor Day, May 1–4) in 2019 (*n* = 370, response rate 93.78%). While the visitors relaxed in the Scenic Area, they were approached by proficient survey administrators and asked to express their own opinions. The sampling frame was stratified by time of day to ensure that sampling events were not biased by daily schedules.

The questionnaire was divided into three parts. Part (1) addressed recreational characteristics and visitor experience. It asked about attitudes toward eleven kinds of activities such as hiking, riding a bicycle, walking, blossom appreciation…. Part (2) addressed value allocation and mapping. The interviewers and respondents engaged in an interactive mapping exercise that entailed visitors to allocate 100 CNY to reflect the importance that they ascribed to each of the 12 social values for the ecosystem services listed in a typology (Table 1), introduced from past research and pre-survey. Following the allocation of preference value points, respondents were asked to identify representative scenic points that embodied the values to which preference points were assigned, using a map of the ELSA created from Google Earth (55). The map of ELSA had a scale of 1:5 km (screen to terrain) and served as a visual basis for communication with respondents. The points marked by respondents were recorded on digital maps for later analysis. Part (3) of the questionnaire asked about visitors' background characteristics. Items concerned gender, occupation, visiting frequency, what attracted them to ELSA. People who reported residence in Wuhan were classified as “residents”; otherwise as “tourists.”

Another data source was Sina Blog, an important platform for people sharing text posts or photos about sightseeing and opinions. Sina Blog has proven to provide an accessible and effective data source (56, 57). It can provide succinct and public geotagged text, link, photo or video content about landscapes that can then be analyzed spatially and quantitatively. Focusing on the ELSA scenic spots identified during the questionnaire phase, we collected 2519 posts about the ELSA from the Sina Blog website across the whole year 2019. Each post includes the main text body and photographs, and the writer's residence and gender. Whether the main body and photos are about the ELSA scenic spots was applied as a sifting condition. Finally, 2165 blog posts for further analysis contained a relevant text description, and there were around 5.5 photos per post on average. The combination of social media and questionnaire were anticipated to provide an interesting perspective on the differences between value preference among residents and tourists, over a meaningful time span.

### Data Processing

Social media photos contain landscape features like lake, trees, greenway, and cherry blossoms, providing considerable information that could help to identify the locations and relevant social value indicators. Adding to the points from the on-site survey, all preferred points allocated to each social value were entered into an ArcGIS geodatabase as a point feature class (*n* = 9971). Without the opportunity to record Sina blog users' perspectives on value allocation, we calculated the average value allocation amount of each social value indicator in the on-site survey, making separate calculations for residents vs. tourists. Specifically, we calculated the average number of points for each value indicator allocated by residents and tourists in the questionnaire phase (Table 2), then applied those calculations when assigning values for the social media data. For example, for a blog from a tourist about Ma'an Mountain Park related to biodiversity, intrinsic, future values, the allocation of each value will be 13.09, 3.53, 8.78 CNY. If this same blog post came from a resident, the values assigned would have been 13.01, 3.55, and 7.14 CNY.

**Table 2:** Statistics of social value for residents and tourists.

**Social value indicators**	**Residents**					**Tourists**				
	**N_COUNT**	**R_RATIO**	**Z_SCORE**	**MAX-VI**	**Average allocation (CNY)**	**N_COUNT**	**R_RATIO**	**Z_SCORE**	**MAX-VI**	**Average allocation (CNY)**
Aesthetic	429	0.19	−32.16	8	13.39	1,065	0.13	−54.48	9	12.44
Biodiversity	218	0.22	−22.09	3	13.01	275	0.27	−23.21	1	13.09
Cultural	332	0.13	−30.17	8	13.16	994	0.08	−55.43	10	15.38
Economic	441	1.00	−36.16	8	4.14	705	0.13	−44.18	4	3.53
Future	345	0.21	−28.14	7	7.15	932	0.12	−51.39	7	8.78
Historic	281	0.10	−28.80	7	9.56	917	0.07	−53.95	7	10.52
Intrinsic	190	0.24	−20.16	2	3.55	238	0.15	−25.05	1	3.53
Learning	247	0.16	−25.26	5	6.36	858	0.08	−51.48	5	4.89
Life Sustaining	238	0.18	−24.14	3	12.19	301	0.20	−26.57	1	11.17
Recreation	542	0.14	−38.43	10	5.79	909	0.12	−50.85	6	6.41
Spiritual	217	0.20	−22.46	2	8.04	295	0.19	−26.60	1	6.12
Therapeutic	116	0.21	−16.23	1	3.66	159	0.28	−17.44	1	4.14

*Number of mapped points (N_COUNT), results of average Nearest Neighborhood statistics (R_RATIO and Z_SCORE), maximum Value Index scores (MAX_VI), average value allocation of on-site survey*.

*Color values represent the magnitude of Value Index, the darker the color, the higher the Value Index, vice versa*.

The geodatabase built for the SolVES process included five environmental characteristics with potential to explain spatial variations in social value intensity (Table 3). The first three environmental characteristics were distance to features relevant to visitor choice in the ELSA, specifically road, water, and elevation. These distances, created using tools available in the Spatial Analyst extension of ArcGIS, reflected the shortest straight-line distance of each cell to features of interest. Next, land use/cover raster data interpreted from ZY-3 (resolution is 5.8 m) was used to represent the natural conditions. Resampling and conversion tools helped to coordinate all of the raster layers at the same resolution (10 m) and extent. Besides, the questionnaire data distributions were tested for normality using the Shapiro–Wilk test in R, version 4.0.0. Data processing also made use of Python 3.8, EXCEL 2019 and ArcGIS 10.5.

**Table 3:** Description of environmental layers and data source.

**Environmental layers**	**Description**	**Source**
Distance to road	Horizontal distance to the nearest road in meters	Deprived from open street map using ArcGIS Euclidean distance tool https://www.openstreetmap.org
Distance to water	Horizontal distance to lake in meters	Deprived from open street map using ArcGIS Euclidean distance tool https://www.openstreetmap.org
Elevation	Digital elevation model (DEM) in meters	NASA Earth data http://vertex.daac.asf.alaska.edu
Land cover	7-class categorical land cover data	Deprived from ZY-3 (5.8 m) remote sensing images

### Analysis of Social Value Indicators and Environmental Layers

**Figure 1:**
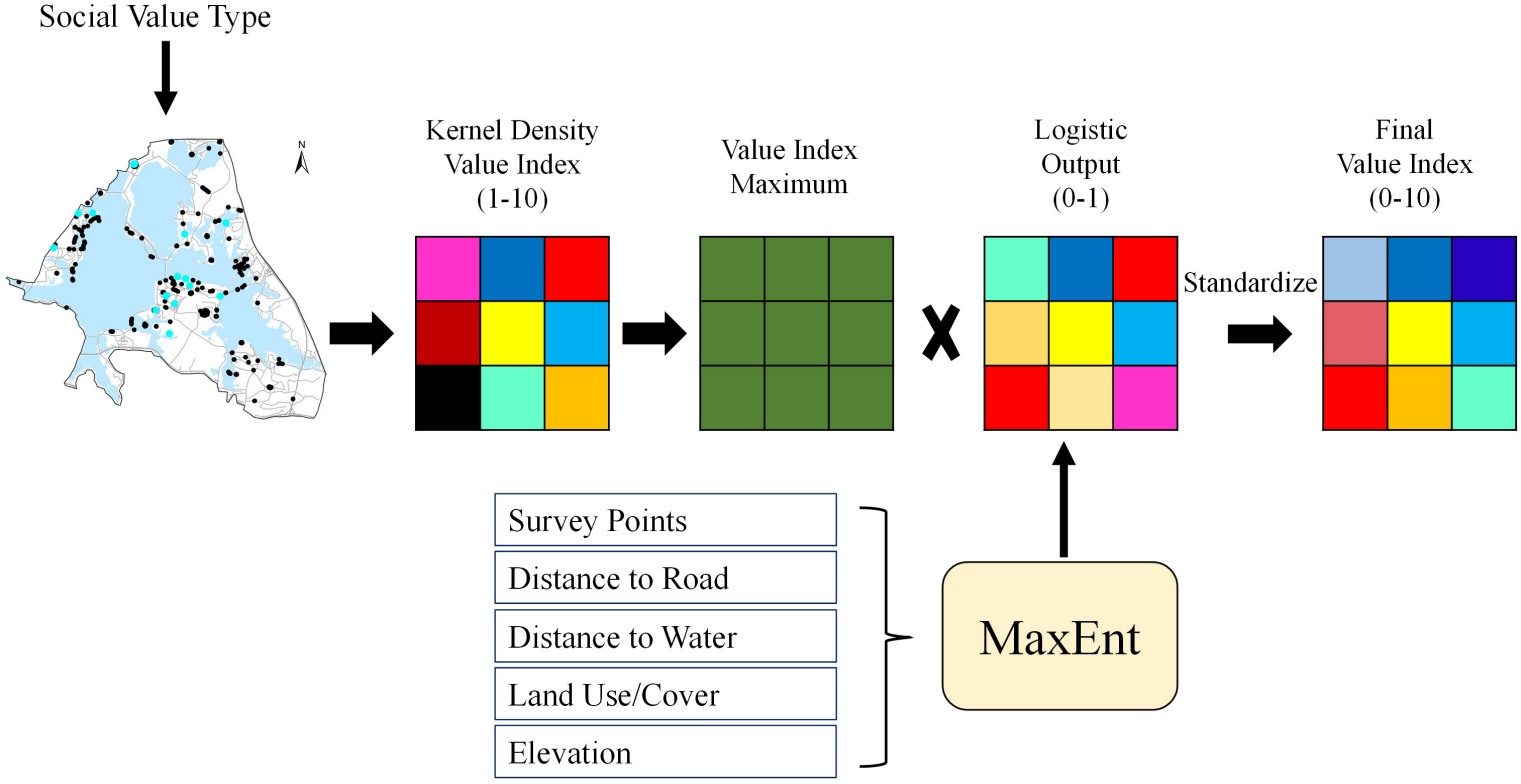
Work flow of data processing in SolVES, adapted from Sherrouse et al. (58).

We identified relationships between mapped social value points and four environmental characteristic layers for both residents and tourists using a GIS mapping application developed by the U.S. Geological Survey (58), Social Values for Ecosystem Services (SolVES 3.0, http://solves.cr.usgs.gov). The composite maps showed social values using a Value Index ranging from 0 (least important) to 10 (most important). We also applied SolVES to create a measure of the density of point features using the Completely Spatially Random (CSR) hypothesis test, which is based on and averages nearest-neighbor statistics. The value *R* represents the ratio of observed distance between points to the expected distance between them; the *Z* score measures how many standard deviations the point is from the mean, and is helpful for determining whether point patterns are dispersing, clustering or random. The weighted kernel density surfaces were generated from the total preference points allocated to each value indicator. All of the surfaces were standardized and normalized to determine the relative importance of each social value (Figure 1).

The Maximum Entropy calculator, MaxEnt, within SolVES generates a logistic surface layer, providing potential locations to which stakeholders can allocate social value. The logistic surfaces generated by MaxEnt predict socially valued locations on the basis of point data that we collected using PPGIS and big-data approaches. The relationship between assigned social values and four primary environmental layers were determined using zonal statistics generated from the integer Value Index (from 0 to 10). The zonal statistics (mean value for continuous data; majority values for categorical data) were compared using independent-sample *t*-tests that were then subjected to Bonferroni tests to neutralize the effects of multiple comparisons.

The accuracy and credibility of the results from the MaxEnt models was evaluated by dividing survey points into “training” and “test” data. MaxEnt parameters were set to reserve 25% of the survey points of social values as test data. The calculation of Area Under the Curve (AUC) in MaxEnt reflected the total area under the Receiver-Operating Characteristic plot (ROC) for the training and test data. Training AUC suggests the goodness-of-fit of the model to the study area, and the test AUC indicates the model's potential predictive capability. We judged our models' fit to the sample data and their predictive potential according to the criteria of Swets (1988): if AUC≧0.90 then the model is deemed good; if 0.90>AUC≧0.70 then the model is useful; and if AUC≦0.70, the model is deemed poor (59).

## Results

### Spatial Patterns of Social Value

**Figure 2:**
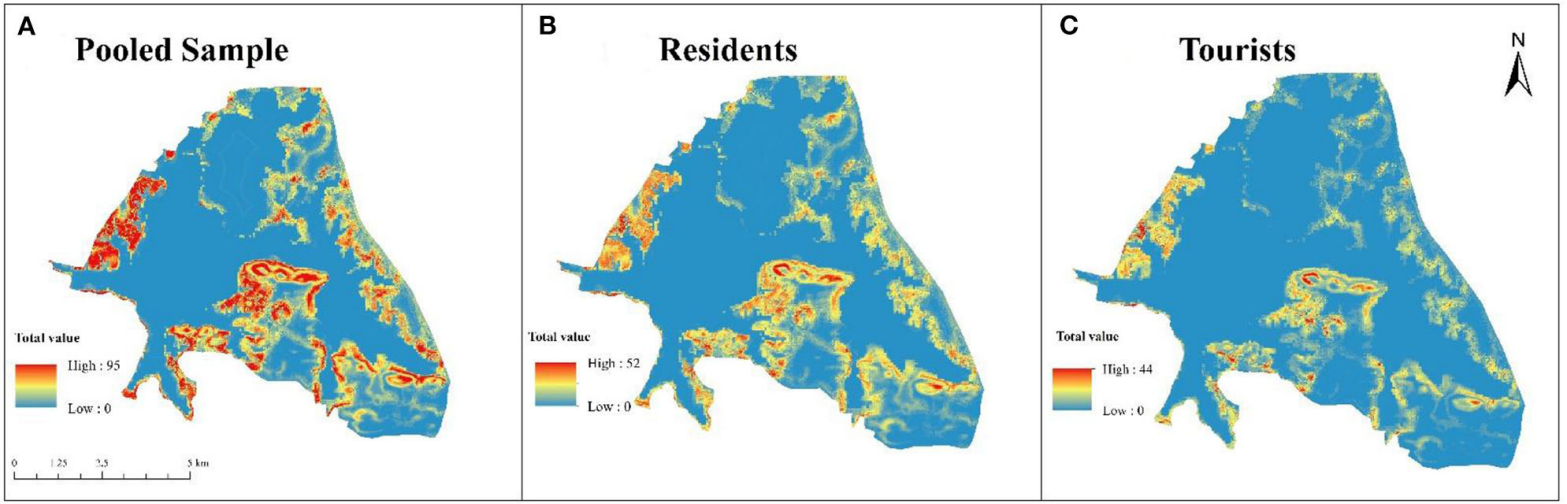
Spatial distribution of total social value at ELSA.

**Figure 3:**
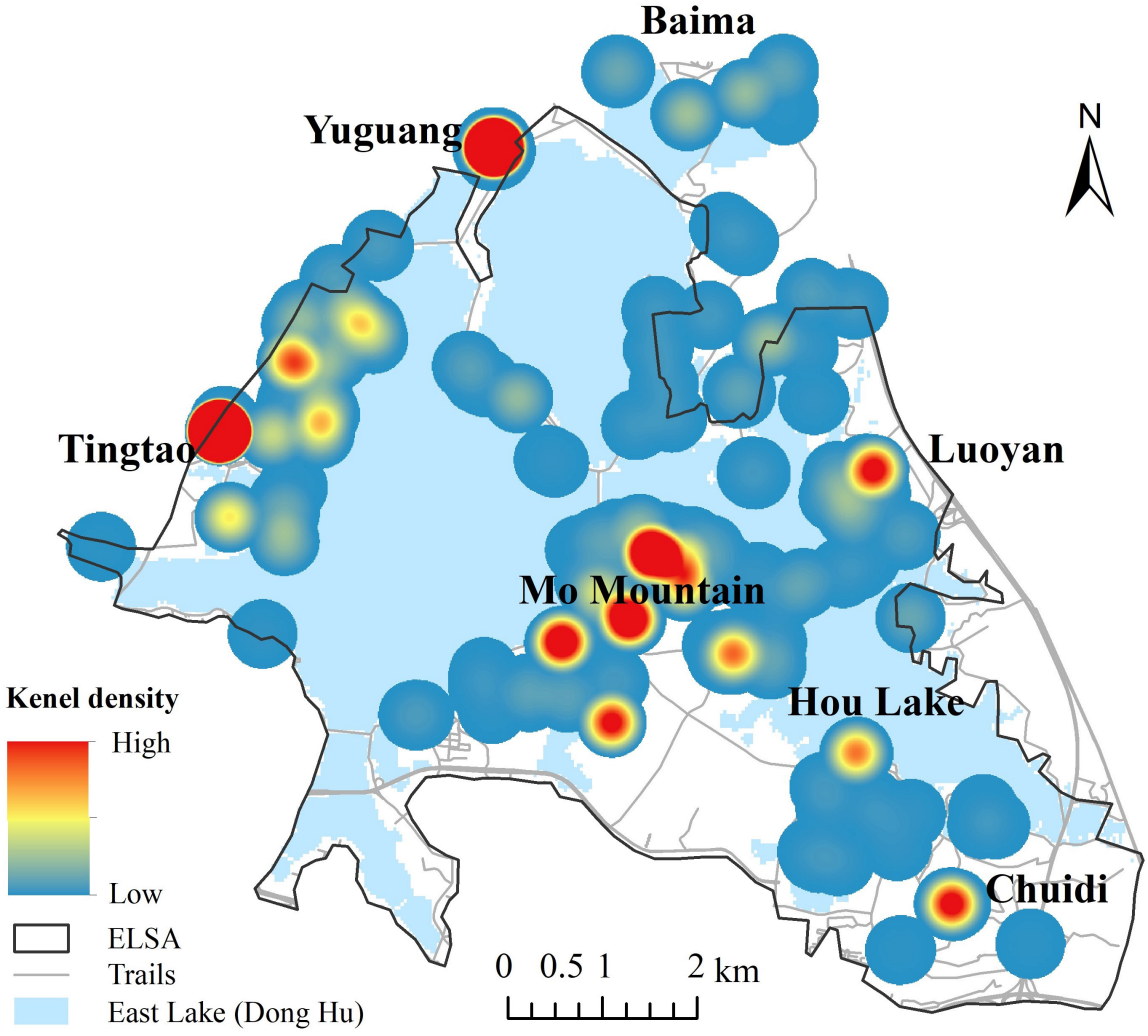
Kernel density of digitalized scenic points at ELSA.

This paper examines the distribution of perceptual social value as its first objective. For the pooled sample, perceived social values are distributed spatially across the whole scenic area with high-frequency clusters at Moshan, Tingtao, and Chuidi districts (Figure 2A). These clusters relate to digitized points at the Hubei Province museum, the botanical garden of the Chinese Academy of Science, Ma'an Mountain Forest Park, the East Lake wetland, and the museum remembering the poet Qu Yuan (Figure 3). Human activities like camping, hiking and cherry blossom appreciation within these locations increase the interaction with nature and thus contribute potential for higher value. Discrepancy between residents and tourists mainly existed in the location and coverage of their hotspots. Inhabitants tended to appreciate scenic points with various social value indicators across a larger geographic gradient spreading over the ELSA (Figure 2B). Inhabitants' assignments of high Value Index clustered in the Moshan and Tingtao districts. In contrast, tourists' distribution of social values encompassed a smaller portion of the ELSA (Figure 2C).

### Disparity of Perceptual Social Value Indicators Between Inhabitants and Tourists

**Figure 4:**
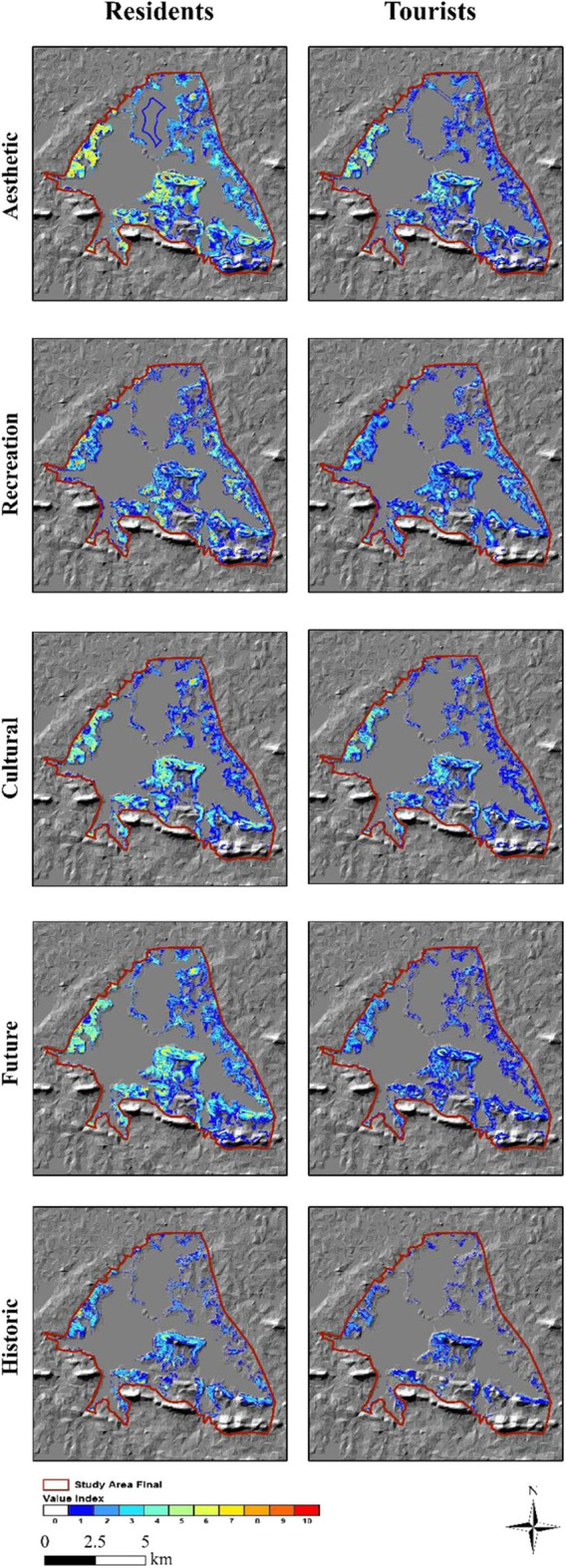
Spatial patterns of five preferable social value indicators.

In response to the second objective, we identified the five most important perceived social values: recreation, culture, history, future, and aesthetic values (Figure 4); the Value Index of each was greater than six (58). To be specific, recreation hotspots at Tingtao, Mo Mountain, Chuidi, Luoyan districts, were the most popular parts of the ELSA. The Value Index assigned by residents (VI = 10) was higher than that assigned by tourists (VI = 6), indicating that recreation value was perceived more important by residents. Hubei Province Museum and the Chutian sightseeing platform embodied cultural value for both inhabitants and tourists. Notably, the cultural value score assigned by residents was higher than that assigned by tourists in the Baima district, probably because its greater distance from the commercial center and inconvenient transport impede visiting from tourists. On the contrary, locations like the National Park Museum, university, and Ancient Stories attract local residents who have better access to these scenic points. Future and history value had the same Value Index (residents: VI = 7, VI = 7; tourists: VI = 7, VI = 7), suggesting that historic evolution and future promotion of the ELSA were considered equally important by residents and tourists. Aesthetic value perceived by tourists (VI = 9) was higher than that perceived by residents (VI = 8), although the spatial distribution was smaller and clustered around Mo Mountain and the Tingtao district, near to subway and bus stations. Residents' ready access over the whole of the ELSA leads to a far-ranging distribution of aesthetic value.

### Relationships Among Preferable Social Values and Environmental Conditions

**Figure 5:**
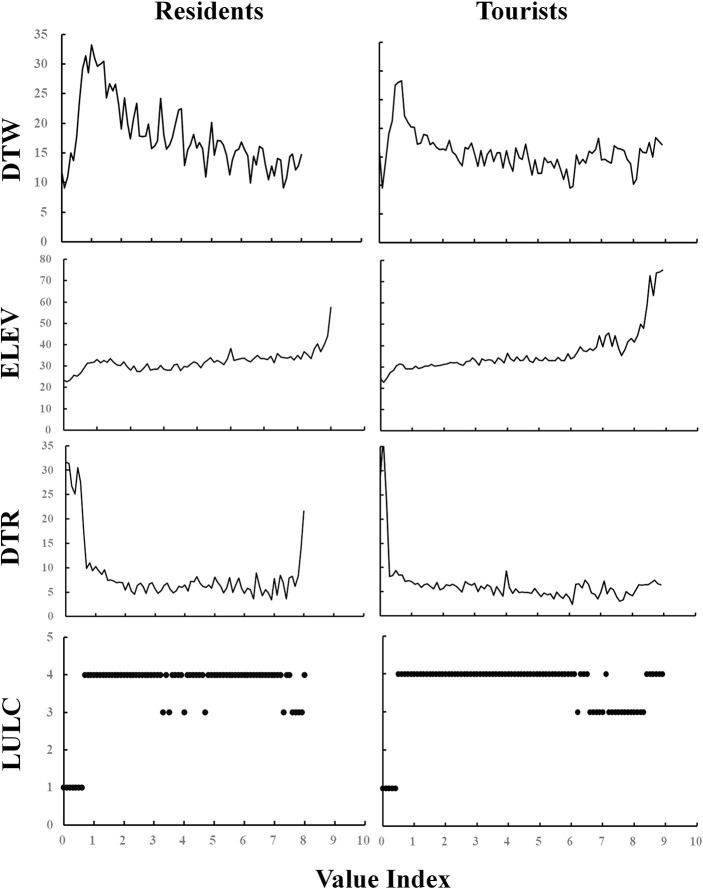
Relationships of aesthetic value and environmental layers. DTR, distance to road; DTW, distance to water; ELEV, elevation; LULC, land use/cover. 1 represents lake, 3 is construction and 4 is forest.

In response to the third objective, relationships among the five important social value indicators and four environmental layers were analyzed. For example, Figure 5 demonstrates the relationship of aesthetic value with primary surroundings. Specifically, the intensity of preferences for aesthetic, cultural, recreation, future, historic values increased as: (1) the distance to road decreased; (2) the elevation increased. As the distance to water increased, aesthetic, future, recreation Value Indexes decreased with delicate differences in the downward trend between inhabitants and tourists. For cultural value, greater distance to water reduced the value recognized by tourists, while cultural value perceived by residents fluctuated. The analysis of relationships with categorical land cover demonstrated similarity between the two stakeholder groups. In detail, the five social value indicators showed lower scores around lake, and higher scores around construction and forest. Furthermore, the contributions of environmental layers to each value differed according to percent contribution (PC). For residents, aesthetic and recreation value were more influenced by distance to water, while cultural, future, historic values were significantly affected by land cover (Appendix Table 1). As to tourists, only recreation value was impacted by distance to water, while the remaining values significantly correlated with land cover. In general, distance to water and land cover exerted more effects on social values. The credibility and accuracy of models were assessed through the training AUC, indicating useful predictive ability (Table 4).

**Table 4:** AUC results for resident and tourist groups.

**Value type**	**Residents**			**Tourists**		
	**Training AUC**	**Test AUC**	**SD**	**Training AUC**	**Test AUC**	**SD**
Aesthetic	0.921	0.909	0.014	0.924	0.918	0.007
Biodiversity	0.867	0.850	0.027	0.881	0.861	0.022
Cultural	0.930	0.935	0.010	0.934	0.951	0.004
Economic	0.940	0.929	0.013	0.933	0.941	0.009
Future	0.912	0.894	0.019	0.928	0.934	0.007
Historic	0.944	0.941	0.014	0.952	0.951	0.005
Intrinsic	0.924	0.934	0.019	0.919	0.940	0.012
Learning	0.906	0.914	0.020	0.939	0.940	0.008
Life sustaining	0.887	0.849	0.020	0.887	0.858	0.019
Recreation	0.918	0.920	0.012	0.914	0.903	0.011
Spiritual	0.899	0.921	0.012	0.899	0.868	0.015
Therapeutic	0.852	0.799	0.025	0.838	0.871	0.020

*SD is the abbreviation of Standard deviation; AUC means area under curve*.

## Discussion

We focused on the disparity of perceived social values associated with ecosystem services for UGS of the ELSA by combining an on-site survey with social media data to provide a better understanding. Comprehensive consideration of social values for ecosystem services at ELSA will be informative to local management and spatial planning. Differing from the monetary evaluation of ecosystem services and geographical investigation, social values of ecosystem services are more suitable for integrating people and natural surroundings into decision-making (60). We classified visitors into urban residents and outside tourists to analyze the perception disparity. These subgroups differed in two aspects: firstly, the spatial distribution and location of social value hotspots; secondly, perceptual importance of social value indicators.

Our results suggest that the spatial pattern of perceptual social values differs between residents and tourists. Residents tended to perceive higher value scores and larger spatial scale of social values than tourists, as a result of constrained transport and familiarity with the scenic spots (26). Our results indicate an urgent need for decision-makers to target spatial planning, for instance, strengthening the accessibility and connectivity among sightseeing spots to facilitate tourist circulation, and extending fundamental service infrastructure for inhabitants. Differences were also found in the social value indicators. Recreation and economic values were more important to inhabitants than tourists, conversely the perception of cultural and aesthetic values. Given that the ELSA makes a difference in offering job opportunities and local economic development, it is not surprising that residents would like to pay more attention to recreation and economic value. Cultural and aesthetic values were rated higher by tourists, suggesting that beautiful scenery of natural and humanistic environment were considerable attractions to visitors.

As for value indicators, aesthetic value appreciated by stakeholders was in agreement with previous studies, because the visuality of scenic spots directly related to the reported perceptions (61, 62). The amusement park and aquarium enhanced the recreational value of the ELSA. Cultural, future and historic values were often collocated in places like the Hubei Province Museum. Future and historic value were equally valued by tourists and residents, which was in accordance with the Chinese construction of ecological civilization and Sustainable Development Goals (SDGs) appealing to sustainability. Residents and tourists share common characteristics in perceptual social values of UGS, suggesting that measures to improve the biodiversity, entertainment facilities, and cultural identity of the ELSA would enhance the relevant perceived social values.

Relatively less important social value indicators, including learning, biodiversity, life-sustaining, intrinsic, spiritual and therapeutic values obtained lower scores. The reason might relate to these values being less tangible and effable. However, life-sustaining and biodiversity values represent the ecological quality of the natural environment, and people benefit from spiritual and therapeutic experiences. Thus, we have emphasized prioritizing preferable values and also taking those neglectable, but fundamental, values into consideration.

Regardless of survey subgroup, the Value Index of social values like aesthetic, future and recreation were higher and closer to roads and water, and at higher elevations. Among the four environmental layers, distance to water (DTW) contributes around 50% to the social value score (Appendix Table 1). This can be attributed to the lake surface occupying 37.5% of the total area. Land use/cover is another important layer for aesthetic, cultural, future and historic values, indicating that landscape composition influences the perceived social values. Elevation contributes about 20% to social values for residents and tourists alike. Since the trail network provides adequate access to every corner of the ELSA, distance to road (DTR) did not exert a significant contribution to social value indicators except for economic values. Spatial planning for increasing opportunities to approach water and vegetation coverage will hence strongly foster social values.

Disparities between residents and tourists mainly exist in the proportion contribution of environmental layers. Taking aesthetic value as an example, distance to water takes up to 60.2% and is the primary influential layer from the perspective of residents. For tourists, distance to water and land use/cover contribute to 34.7 and 37.7%, respectively. Large water surface is propitious to local climate and temperature regulation. Besides, camping and boat sailing are representative activities of residents relating to water. Tourists equally appreciate both water and landscapes in aesthetic value. Perceived aesthetic value will therefore be promoted by the addition and improvement of facilities near water. And better allocation of land cover exerts positive effects on tourists larger than that on residents.

On-site survey and social media data have become the main source of data collection in the evaluation of social value for ecosystem services (63, 64). The advantages of on-site surveys can be found in detailed publications about respondents and empirical methods (65), although survey administration is limited by time and economic cost, and also by spatial and temporal restrictions (e.g., investigation of remote natural areas and historical situations) (66). Social media compensates for these disadvantages and is recognized as a free, fast and useful source of data (67). We attempted to combine the advantages of both kinds of data in this research. The results suggested that the on-site survey compounded well with social media, for analysis using the SolVES tool. A longer-timespan data source could provide abundant temporal information, potentially applicable to dynamic analysis of ecosystem services over a time cycle. Different data sources offer ways to develop a more comprehensive knowledge of social values of ecosystem service for UGS.

## Conclusions

This research combined environmental characteristics with subjective perceptions of visitors to quantify, assess and map social values across the East Lake Scenic Area. We combined on-site survey responses with social media data into a comprehensive knowledge-base of location-associated social values, allowing an analysis of how social values relate to environmental characteristics, and how those associations differ between inhabitants and tourists. Results demonstrated that the spatial pattern and Value Index of social values could be associated with surrounding layers. Residents and tourists differed in distribution of social value, the importance of value indicators, and which environment layers were most influential to their experiences. Integrating different data sources extends the data acquisition approaches available for the SolVES model, which could potentially visualize the temporal dynamics of social values in the long run. Moreover, this study emphasized the social value discrepancies between residents and tourists to provide insight into how demands of different stakeholder groups can be incorporated into urban planning and green space management processes. Similar studies in other regions of the world are necessary to examine how different data sources and different stakeholders' categories could contribute to urban green space planning.

Two limitations need to be addressed in this study. Firstly, we conducted the on-site survey only once during 1st−4th May, spring of 2019. People's perceptions in every season or month should be surveyed for the purpose of coordinating with the whole year's social media data. Additionally, on-site surveys and social media data collection are subject to sampling bias. Because young adults are the main users of Sina Blog, opinions of young children and aged people are less considered (68). Future research should consider a fuller range of age categories to provide more comprehensive understanding.

## Data Availability Statement

The raw data supporting the conclusions of this article will be made available by the authors, without undue reservation.

## Ethics Statement

Ethical approval for this study was not required in accordance with local legislation and national guidelines.

## Author Contributions

YC: designing, writing the manuscript, and data processing. XK: revising the manuscript. MM: designing the structure and revising the manuscript. PC: data processing. All authors contributed to the article and approved the submitted version.

## Conflict of Interest

The authors declare that the research was conducted in the absence of any commercial or financial relationships that could be construed as a potential conflict of interest.
